# Determination of Parameters for the Supercritical Extraction of Antioxidant Compounds from Green Propolis Using Carbon Dioxide and Ethanol as Co-Solvent

**DOI:** 10.1371/journal.pone.0134489

**Published:** 2015-08-07

**Authors:** Bruna Aparecida Souza Machado, Gabriele de Abreu Barreto, Aline Silva Costa, Samantha Serra Costa, Rejane Pina Dantas Silva, Danielle Figuerêdo da Silva, Hugo Neves Brandão, José Luiz Carneiro da Rocha, Silmar Baptista Nunes, Marcelo Andres Umsza-Guez, Francine Ferreira Padilha

**Affiliations:** 1 Institute of Research and Technology, Tiradentes University, Aracaju, Sergipe, Brazil; 2 Faculty of Technology, SENAI/CIMATEC, National Service of Industrial Learning–SENAI, Salvador, Bahia, Brazil; 3 Faculty of Pharmacy, State University of Feira de Santana, Feira de Santana, Bahia, Brazil; 4 Biotechnology, Federal University of Bahia, Salvador, Bahia, Brazil; College of Agricultural Sciences, UNITED STATES

## Abstract

The aim of this study was to determine the best processing conditions to extract Brazilian green propolis using a supercritical extraction technology. For this purpose, the influence of different parameters was evaluated such as S/F (solvent mass in relation to solute mass), percentage of co-solvent (1 and 2% ethanol), temperature (40 and 50°C) and pressure (250, 350 and 400 bar) using supercritical carbon dioxide. The Global Yield Isotherms (GYIs) were obtained through the evaluation of the yield, and the chemical composition of the extracts was also obtained in relation to the total phenolic compounds, flavonoids, antioxidant activity and 3,5-diprenyl-4-hydroxicinnamic acid (Artepillin C) and acid 4-hydroxycinnamic (p-coumaric acid). The best results were identified at 50°C, 350 bar, 1% ethanol (co-solvent) and S/F of 110. These conditions, a content of 8.93±0.01 and 0.40±0.05 g/100 g of Artepillin C and p-coumaric acid, respectively, were identified indicating the efficiency of the extraction process. Despite of low yield of the process, the extracts obtained had high contents of relevant compounds, proving the viability of the process to obtain green propolis extracts with important biological applications due to the extracts composition.

## Introduction

Propolis is a resinous material collected by bees from sprouts and barks of different plants and trees. It is originally used as a substance of defence for the hives [1–3]. Propolis has a pleasant aromatic odour and its colour varies from green-yellow, dark brown to red, depending especially on the vegetable source, season and its geographical origin. *Baccharis dracunculifolia* DC (Asteraceae) is a native plant from Brazil and it is considered the most important botanical source of green propolis, predominant in the Brazilian southeast [4]. Different studies have demonstrated the biological properties of propolis extracts such as its anti-oxidant action [5–6], antimicrobial action [7–9], anti-inflammatory action [10–11], anti-parasite action [12–13], immune-modulatory action [14–15], anti-tumor action [16–19], antiviral action [20–21] and others [22–23]. These biological activities are especially attributed to the phenolic acids, flavonoids, terpenes and sesquiterpenes present in the propolis [24–26].

The physiological and biochemical mechanisms responsible for the biological effects of propolis are still to be determined. The majority of the therapeutic effects, however, were suggested through an association between its anti-microbial actions and the capacity to sequestrate free radicals [27–28]. Furthermore, the anti-oxidant and anti-inflammatory effects have been extensively attributed to its high flavonoid content [29]. One of the main phenolic acids presents in samples of green propolis is the 3,5-diprenyl-4-hydroxicinnamic acid (HPPC), also known as Artepillin C. This compound is particularly relevant for the pharmaceutic industry, considering its preventive and anti-tumoural effects *in vitro* and *in vivo*, identified in some studies [2, 30–34].

Considering the different types of processes used around the world to obtain propolis extracts, ethanol is the first choice of solvent, especially due to the affinity of its chemical characteristics with the matrix. Other solvents such as ethylic ether, water, methanol and chloroform can also be used for the extraction of specific classes of propolis constituents [35–36]. The extracts can be obtained through conventional techniques such as extraction by Soxhlet, maceration, or alternative methods, such as extraction with supercritical fluid [24–25,37–39]. Supercritical fluid extraction (SFE) shows very desirable characteristics, considering its high flexibility and it can adjust the solvent power and process selectivity. Besides, the high quality of the product obtained, when compared to the conventional methods, shows more advantages due to low use of polluting organic solvents. In the last decades, SFE has been widely used for the extraction of scents, fragrances, as well as active constituents, especially from vegetable matrices [40]. Supercritical fluids have low viscosity as gas, high density as liquids, and an intermediate level of diffusion somewhere between gases and liquids, varying with its density [41–43]. Carbon dioxide is the most widely used solvent due to its low cost, easy available in high purity levels, non-toxic, non-flammable and non-explosive. Another advantage is that the carbon dioxide is a gas at room temperature and pressure, also easily removed after the extraction process [44].

Analysing the great relevance given to the SFE technology as an alternative extractive process, and the importance and promising biological effects of the Brazilian green propolis, the aim of this work was to define the best conditions to the process. This includes establishing the temperature, pressure and co-solvent concentration necessary for the extraction of green propolis, using carbon dioxide as a supercritical fluid. In this sense, the global curves of extraction, yield and global extraction isothermals were defined by the compounds studied, as well as the characterization of the extracts obtained, related to some components: 3,5-diprenyl-4-hydroxicinnamic acid (Artepillin C) and acid 4-hydroxycinnamic (p-coumaric acid).

## Materials and Methods

### Obtainment and processing propolis

Approximately two kilos of green propolis were donated by the company Apis Nativa Produtos Naturais (Prodapys–Santa Catarina–Brazil), originated from the south of the Paraná state, Brazil. The sample of green propolis was grinded in a mill (Cadence–Brazil) and then sieved, aiming to obtain an adequate particle size (diameter 52–92 μm) to increase the surface area and homogeneity of the starting material in the extraction bed. Small quantities of propolis (100 g) were kept at -10°C, in bottles protected with aluminium foil in inert atmospheric conditions (N_2_), in order to avoid degradation of the material.

### Materials and reagents

Ethanol (HPLC degree) and acetic acid (HPLC degree) were obtained from Merck Co. (Darmstadt, Germany) and methanol (PA) from Sigma-Aldrich Chemical Co. (St. Louis, MO, USA). A cellulose ester membrane filter of 0.45 μm (SLCR025NS, Millipore Co., Bedford, Massachusetts, USA) was used. The carbon dioxide (CO_2_) used in the extraction had 99.9% purity (White Martins Industrials Gases–São Paulo, Brazil). The standard 3,5-diprenyl-4-hydroxicinnamic acid (Artepillin C–cas number 72944-19-5) was acquired from Wako Pure Chemical Industries, Ltd. (Osaka, Japan) and the acid 4-hydroxycinnamic (p-coumaric acid–cas number 501-98-4), 1,1-diphenil-2-picrilhidrazil (DPPH), Acid Gallic (cas number 149-91-7) and Quercetin (cas number 117-39-5) were acquired from Sigma-Aldrich Chemical Co. (St. Louis, MO, USA).

### Characterization of raw material

The determinations of humidity, protein and total ash contents were made according to the official methods of Association of Official Agricultural Chemists (AOAC) [45]. The total lipids were extracted and quantified through the cold extraction method described by Bligh & Dyer [46]. The determination of the mineral content was made in a digital flame photometer (DM-62, DIGIMED, São Paulo—Brazil) and the fibre content was obtained through the automatic fibre analyser (A-220, ANKON, New York–USA) [47]. The quantification of the water activity took place using a decagon LabMaster (Novasina, Lachen–Switzerland), with electrolytic cell CM-2. The analyses were performed in triplicate. The Scanning electron microscopy (SEM) was performed in a scanning electron microscope JEOL JSM-6390LV (USA). After drying in an oven (105°C/45 min), the sample of crushed propolis was fixed manually using a tweezer (PELCO Tweezers) of aluminum metal surfaces covered with carbon double-sided tape, called stubs. Because of the need for interaction of the electron beam with the sample, it was performed by coating deposition of metallic gold ions (sputtering). The sample was metalized in gold in a “Sputter oater” from Balzers, model SCD 50 (20nm). Then the stubs containing the metallic samples were stored in plastic boxes (storage boxes), duly sealed with parafilm (PARAFILM M) to prevent moisture absorption. After 24 hours of rest, the samples were analyzed at different magnifications (Voltage 12 kV, Working Distance 12 mm, Spot size 44, Vacuum Mode HV).

### Extraction Procedure

The equipment used to obtain the extracts of green propolis was a pilot unity called SFT-110 Supercritical Fluid Extractor (Supercritical Fluid Technologies, Inc.), composed by a high pressure bomb (capacity up to 10,000 psi), extraction cell (capacity 100ml), oven (with a pre-heater), static/dynamics valve and restrictor valve, flow meter and CO_2_ cylinder (S1 Fig). A CO_2_ cylinder with fishing tube was used to ensure that only CO_2_ in liquid state was used in the system, a requirement demanded by SFT-110. The cell was assembled in the oven and kept at the pre-selected temperature. The temperature of the restrictor valve was adjusted to 80°C for all extraction processes and 50°C for cleaning. The extract was collected in a 50 ml glass vial, immersed in ice at room temperature and the total of CO_2_ was measured using a flow totalizer (ITRÓN, ACD G1.0, Argentina). The output of CO_2_ in the system was of 6.0 g/min in all experiments.

The extraction processes were described graphically, by the curves obtained through the analyses of the extracts yield over time, using extraction kinetics and the isotherms obtained. The definition of the best extraction conditions consisted of three phases, described below. All experiments were performed in triplicates.

#### Phase 1 – Determination of the extraction global curve and S/F (CO_2_ mass/propolis mass)

The pilot kinetics of extraction was performed in the mildest of temperature and pressure (100 bar and 40°C) using 7.5 g of sample and CO_2_ flow of 6 g/min. At pre-determined periods, the collection bottles used for extraction were substituted for clean ones previously weighted, until no more extraction was being yielded. The extract mass contained in each bottle was measured using an analytical scale (SHIMADZU—São Paulo, Brazil), and thereafter the accumulated yield of the extract versus the corresponding accumulated S/F was represented in a graph, in order to observe the system behaviour. The S/F was calculated as the rate between the total mass of the solute (considering the volume of CO_2_ in the system and its density) and the mass of feed on a damp base (7.5 g) [48]. The experiment lasted until the bed was considered exhausted, and 13 experiments in each assay were obtained. The total mass of the extract was determined as the addition of the extract obtained during extraction and the extract recovered in the cleaning process. The cleaning process consisted in recovering the extract inside the tube line that conducts it out of the extraction recipient with CO_2_ and ethanol. The glass bottles (used in the processes, extraction and cleaning) were purged with N_2_ and stored at -10°C until analysis. The global yield was calculated by the rate between the extract mass (Mextract) obtained and the propolis mass (Mpropolis) used to make the bed. The S/F adopted for the subsequent extractions was determined by the analysis of the obtained graph. For each collection bottle, in addition to order the yield of extraction, the content of total phenols and anti-oxidant activity were also determined.

#### Phase 2 – Determination of co-solvent percentage

In this phase, extractions were performed in triplicates, using 1% and 2% ethanol in relation to the CO_2_ mass used in the process. These experiments were performed in conditions of 50°C, 250 bar and flow of CO_2_ at 6 g/min [2,24–25,28,38,49]. The ethanol was homogenised with the sample and placed in the extraction cell, together with wool and glass pearls to fill the cell (S2 Fig). The adopted S/F was that obtained from the pilot kinetic at the previous phase (S/F = 110). The choice of percentage for the co-solvent adopted for the extraction of active compounds from propolis was determined through the characterization of the extracts (total phenols, anti-oxidant activity and flavonoids) and the quantification of p-coumaric acid and Artepillin C.

#### Phase 3 – Global yield isotherms (GYIs)

The GYIs were elaborated aimed at identifying the ideal conditions of pressure and temperature for extraction of the studied matrix. They were determined at 40 and 50° C and 250, 350 and 400 bar [48,50]. The extraction conditions were those determined in previous phases (S/F = 110, 1% co-solvent and total extraction time of 2 h 30 min). The extraction cell consisted of 7.5 g green propolis (homogenised in co-solvent), wool and glass pearls packed together to avoid the preferred paths of CO_2_ and totally fill the bed. The supercritical extraction process occurred using the desired pressure in the system, keeping the system pressurized for 30 minutes, followed by opening the valve and collecting the extract for 2 hours (S/F = 110) [48] (S3 Fig). The extracts of each assay were characterized by analysing the total phenol content, total flavonoids, anti-oxidant activity (IC_50_), p-coumaric acid and Artepillin C.

### Chromatographic Analysis

Solutions of 10 mg/ml of propolis extracts obtained in the different conditions of the process were prepared and dissolved in ethanol and placed in ultrasound bath (Sanders, SONICLEAN 6 –Minas Gerais, Brazil) for 30 minutes (Electronic timer microprocessor–Temperature 35°C electronically controlled and Ultrasound frequency 40 kHz). The exposure to the ultrasound system only started after reaching 35°C. The samples were filtered in a cellulose ester membrane filter 0.45 μm (Millipore) for subsequent injection in the high performance liquid chromatograph (HPLC).

The chromatographic experiments were performed with the system HPLC EZChrom Elite, consisting of a VRW HITACHI L-2130 pump, equipped with an automatic injector and diode arrangement detector (DAD) VRW HITACHI L-2455 and oven VRW HITACHI L-2300. The chromatographic separation was based in the method proposed by Daugsch [51], adapted. The column LiChroCART Purospher StaR RP18-e (75 mm x 4 mm i.d.) (3 μm) (Merck, Darmastad, Germany) was used together with a pre-column LiChroCART 4–4 LiChrospher 100RP18 (5 μm) from Merck.

**Fig 1:**
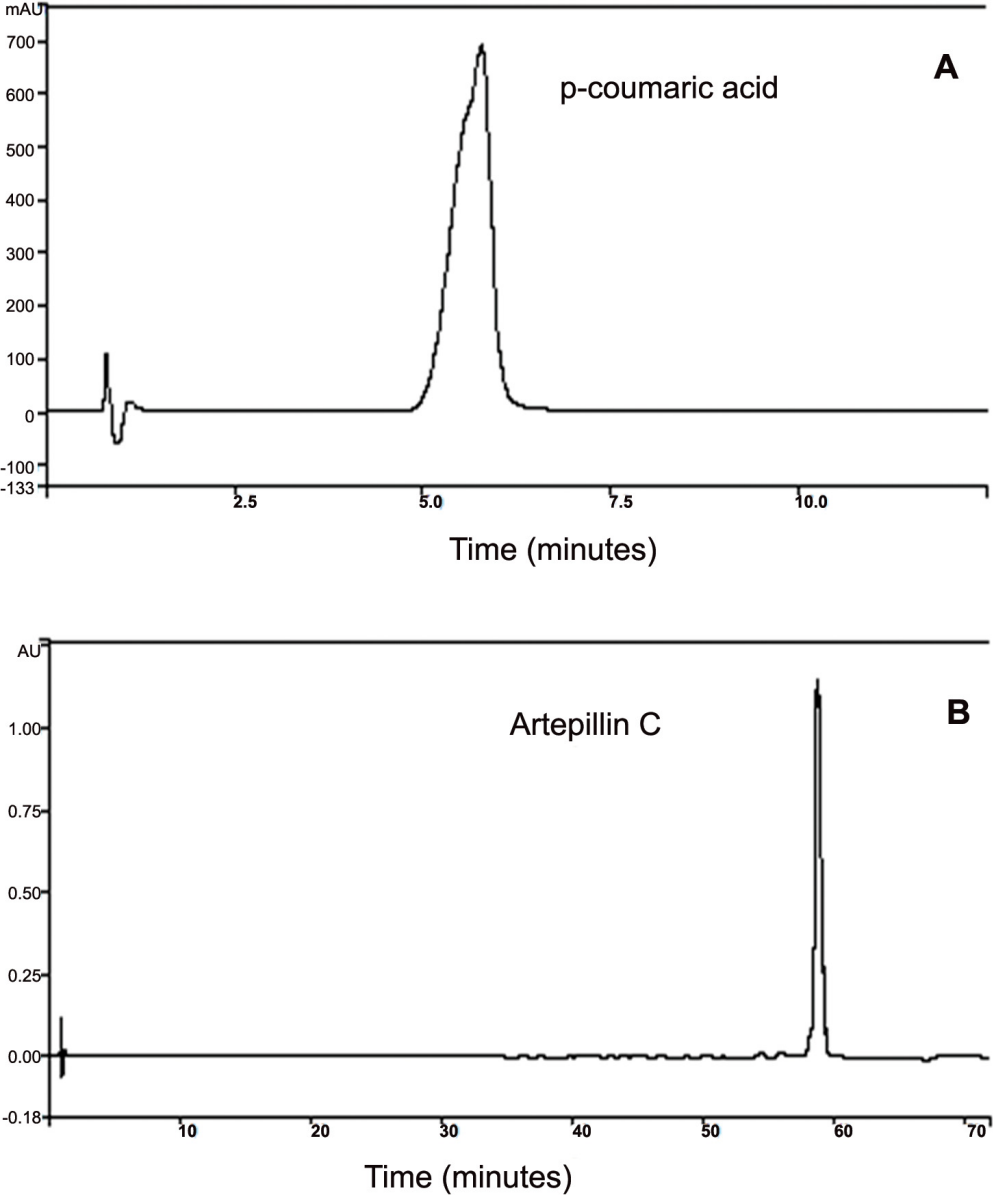
Chromatograms obtained to the p-coumaric acid and Artepillin C standards at 290 nm by HPLC.

The conditions for analysis were performed with an elution gradient with a mobile phase of acetic acid 5% (aqueous phase) and methanol (organic phase) in different proportions and with total analysis time of 70 minutes (0 min—80:20; 10 min 70:30; 15 min—60:40; 30 min—50:50; 45 min—40:60; 60 min—30:70; 65 min—0:100; 70 min—80:20). The volume of injection was of 10 μL. The equipment was operated at room temperature (25±2°C). The reading of the diode arrangement detector was in the range of 200 to 400 nm and the chromatographic acquisition was defined at 290 nm. The identification of the compounds was performed through the comparison of time of retention and ultraviolet spectrum between the samples and the controls (standard) (S4 and S5 Figs). In the Fig 1 are illustrated the chromatograms obtained to the standards analysed. Aiming at ensuring the reliability of the results obtained, a validation took place according to the National Health Surveillance Agency (ANVISA) [52] and National Institute of Metrology, Standardization and Industrial Quality (INMETRO) [53] methodologies. This was done in accordance to the parameters of selectivity, linearity, precision, accuracy, detection limits and quantification limits.

### Determination of the Total Phenolic Compounds

The total phenolic content was determined using the Folin Ciocalteau reagent [54–55]. The reaction was prepared with a 0.5 ml aliquot of propolis extract (dissolved in ethanol aimed at obtaining a concentration of 200 μg/ml), 2.5 ml aqueous solution of Folin-Ciocalteau 10% and 2.0 ml sodium carbonate at 7.5%. The mixture was introduced in a thermo-regulated bath at 50°C for 5 minutes; afterwards, the absorbance was measured in a spectrophotometer LAMBDA 25 UV/vis Systems (PerkinElmer, Washington—USA) at 765 nm.

The quantity of total phenolic was expressed as Gallic acid equivalents (g of Gallic acid (GAE) in 100 g) through a calibration curve (y = 0.0073x – 0.066 R² = 0.9995) using known solutions to Gallic acid standard in the same conditions (λ = 765 nm). The Folin Ciocalteu method is associated to the appearance of a blue colouring due to the oxidation of phenols in basic medium [56].

### Determination of Flavonoid content

The determination of flavonoid content was performed through the reading in a spectrophotometer (LAMBDA 25 UV/Vis Systems—PerkinElmer USA) at 415 nm, using aluminium chloride at 2% in methanol [57] in a 1:1 solution (extract:aluminium chloride). The same procedure was performed using known solutions of quercetin standard to elaborate a standard curve (y = 0.0276x – 0.0256 R² = 0.9996). The quantity of total flavonoids was expressed as quercetin equivalents (g of quercetin (GQ) in 100 g).

### Determination of Anti-oxidant Activity in vitro (1,1-diphenil-2-picrilhidrazil–DPPH)

The anti-oxidant activity in vitro of propolis extracts obtained in different conditions was evaluated using the reactive 1,1-diphenil-2-picrilhidrazil (also known as the capacity to sequestrate the radical DPPH) [58–59]. Five dilutions of the extracts were prepared (20 to 400 μg/ml) in triplicates. An aliquot of 1 ml of each extract dilution was transferred to assay tubes with 3.0 ml of the ethanoic solution (95%) of the radical DPPH (0.004%). After 30 min incubation in the dark and at room temperature, the reduction of the free radical DPPH was measured through the reading of absorbance in 517 nm (calibration curve y = 0.1897x – 4.5 R² = 0.9955) (spectrophotometer LAMBDA 25 UV/Vis Systems–PerkinElmer, Washington—USA).

The same procedure was adopted with ethanol instead of the sample, considered white. The capacity to sequestrate free radicals was expressed as the percentage of oxidation inhibition in the radical and calculated according to Equation 1. The IC_50_ value (necessary concentration of the extract to sequestrate 50% of DPPH radical) was calculated through the line equation based on the concentrations of extracts and its respective percentages of radical DPPH sequestration.

$$\%\;sequestration=100-\frac{\text{final absorvance of sample x}\;100}{\text{white absorvance}}$$ (1)

### Statistical Analysis

The results found were evaluated using analyse of variance ANOVA (one-way) and the Tukey Test, in order to identify whether the alterations in the parameters evaluated were significant at 95% confidence.

## Results and Discussion

### Characterization of raw propolis

The green propolis evaluated in this study presented a physical-chemical profile of 7.13±0.12% for humidity, 3.05±0.03% for total ash, 9.98±0.83% for proteins, 45.75±1.71% for lipids and 20.89±1.39% for fibres. There are very few works reporting the centesimal characterization of propolis. However, the values obtained in this study are similar to those found in the literature for other samples of propolis, which presented variations of 3.4–7.7% for humidity, 1.6–4.4% for total ash and 6.5–32.3% for lipids [25,60–65]. The variations found for the centesimal profile could be due to the type of propolis studied, geographical region, environmental conditions, and even to the collection period [64,66–68]. It should be highlighted that the centesimal composition of the studied sample is in agreement with the specifications established by the Brazilian legislation [69], as well as the legislations of other countries (Argentina and Switzerland). Regarding the water activity (Aw), the sample showed a value of 0.704±0.008, considered as a product with intermediate Aw. The Aw is considered a parameter totally linked to the humidity of the product, which allows the determination of its capacity for conservation, microbial propagation and occurrence of chemical reactions [70]. From the ash obtained, a mineral composition was performed in relation to the contents of sodium, potassium, lithium and calcium. The values found were 3.00±0.01, 331.70±5.81, 1.80±0.01 and 9.00±0.01 mg/Kg, respectively. The main micro-elements present in propolis samples are aluminium, calcium, strontium, potassium, iron, copper and manganese [1]. The determination of the percentage of micro and macro nutrients in propolis samples have a great importance to the nutritious knowledge and calorific value of diet in different animals, such as fish, chicken and milk cows [65,71–72].

**Fig 2:**
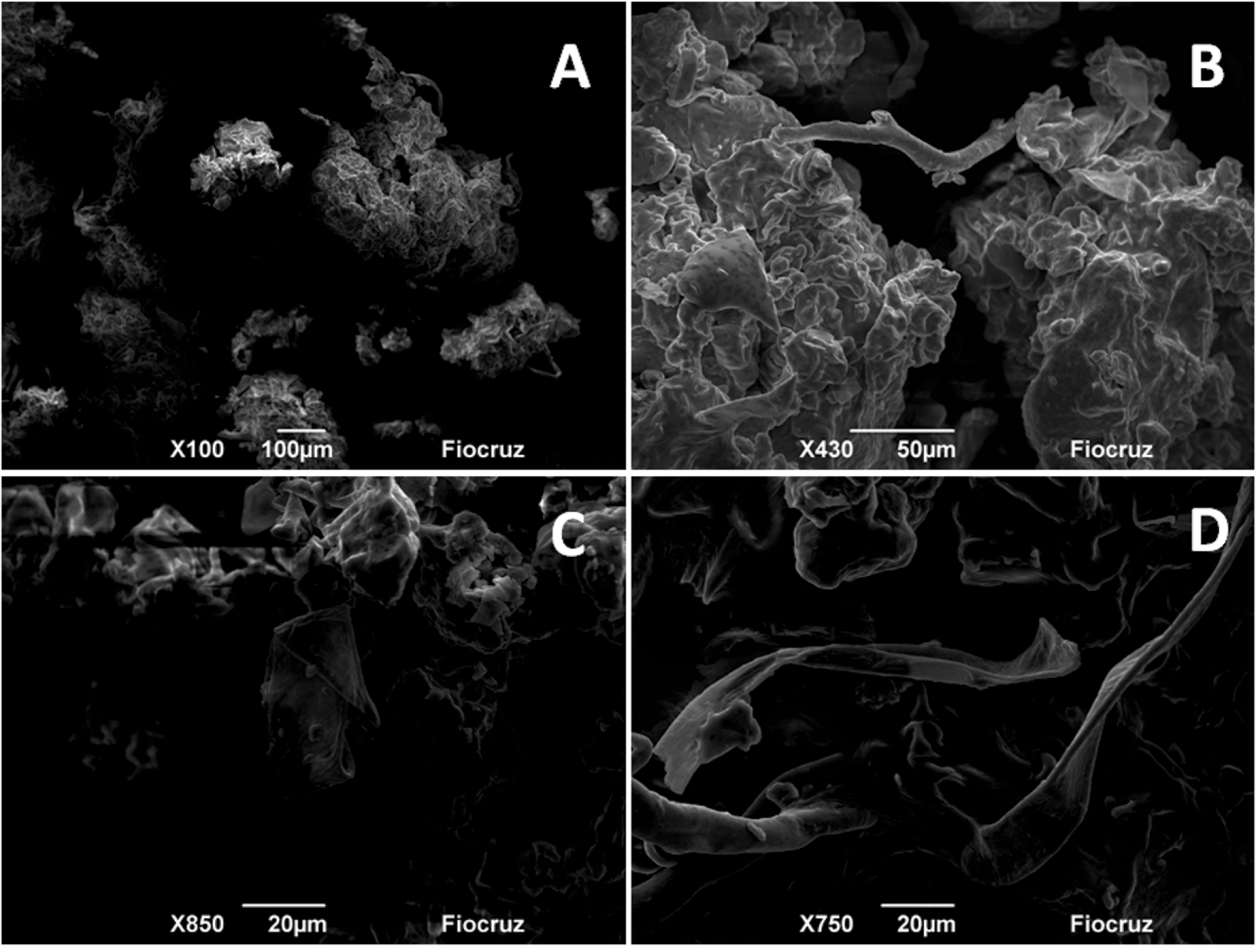
Images obtained by Scanning electron microscopy (SEM) for the sample of green propolis (Zoom of 100—A, 430—B, 800—C and 750—D times).

On Fig 2, there are the micrographs of the green propolis particles used as an extraction material. The majority of the particles are presented in isolated form (Fig 2A), however with a tendency to form big agglomerates (Fig 2C). The same profile was presented in the work of Tylkowski et al., [73] for samples of propolis originated from Bulgaria. In all images, it is possible to observe rugged surfaces covered by layers of wax and extractives, besides revealing the presence of vegetal constituents, probably non-glandular trichomes and/or glandular and resinous substances from vegetative apexes of *Baccharis dracunculifolia* (Fig 2B, 2C and 2D) [4,74–76].

### Determination of the extraction global curve and S/F

Initially, a pilot kinetic of extraction (kinetic curve) was determined, aimed at determining the quantity of solvent (CO_2_) and consequently the time of extraction necessary to reach the diffusional period and to ensure that the global yield assays had results, which were close to the real exhaustion of the extraction bed, maximizing the quantity of extract obtained. For that purpose, milder temperature and pressure than those studied were used (40°C; 100 bar).

In preliminary assays, a 15g mass of sample was used for the extraction process (40°C, 100 bar, 6.0 g/min CO_2_ flux, 15 g propolis) and subsequent obtainment of pilot kinetic curve. However, during the experiment, it was needed to reduce the quantity of samples, considering that after 327 minutes of extraction, there still was a great quantity of extract getting out, which demanded a large expenditure of energy and time to finalize the process. According to Meireles [50], considering that the exhaustive extraction process can take hours of experimental work, the sample mass reduction can influence positively to the lower energy expenditure. The reduction of the sample mass will not influence the selection to determine the S/F and the selection of the best conditions of temperature and pressure

**Fig 3:**
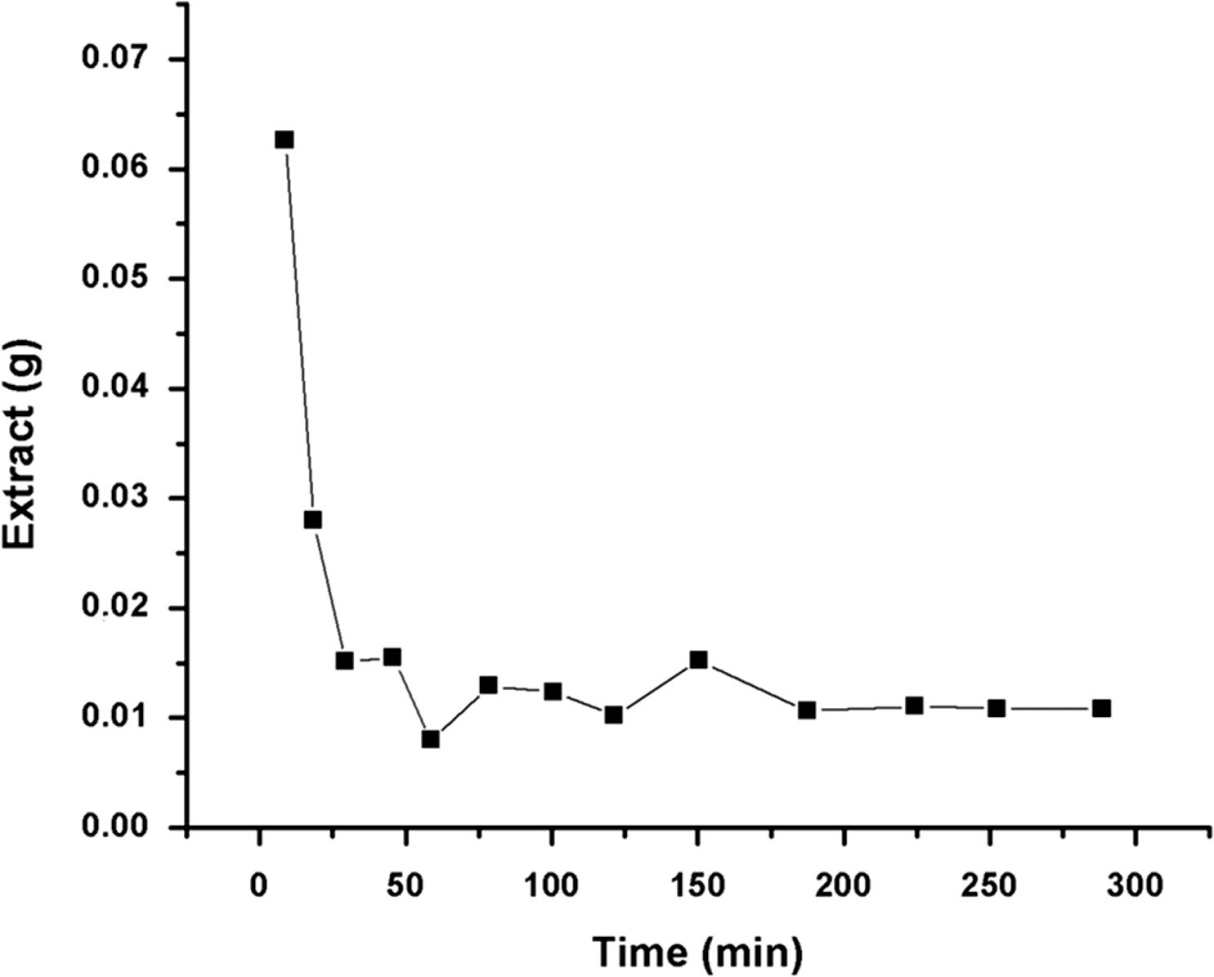
Profile of extraction for green propolis in relation to the extract mass obtained at different times for obtaining the pilot kinetic (Conditions: 40°C, 100 bar, CO_2_ flux of 6 g/min and 7.5 g of green propolis).

**Fig 4:**
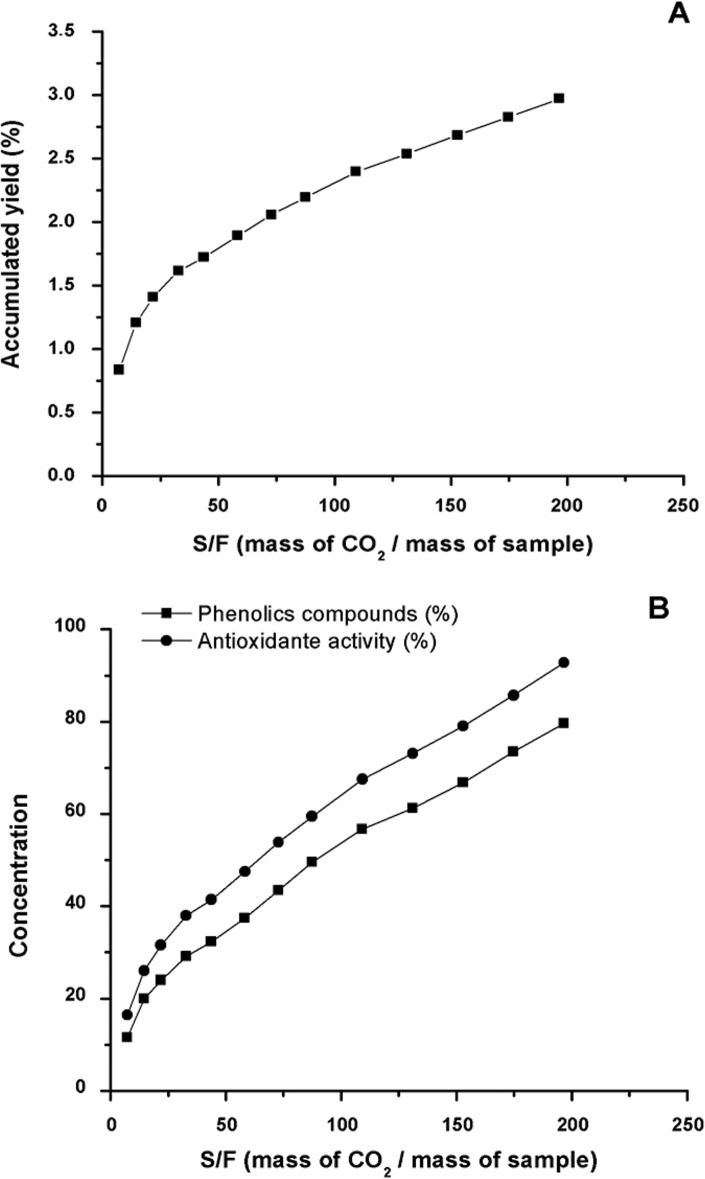
Global Curves of Extract for: (A) yield in mass of accumulated extract; (B) yield of the phenolic compound content and of the antioxidant activity (accumulated values) (Conditions: 40°C, 100 bar, CO_2_ flux of 6.0 g/min and 7.5 g of green propolis).

As mentioned in the methodology, a mass of 7.5 g of green propolis was adopted to determine the extraction kinetic curve. The accumulated yield (extract mass) obtained and its corresponding S/F, as well as the results for the accumulated yield for total phenolic compounds and antioxidant activity (%) are presented in Table 1. On Fig 3, the kinetic curve obtained by the quantity (mass) of extract (values not accumulated) versus time of extraction is presented (S1 Table). On Fig 4, the extraction global curves for yield, phenolic compounds and antioxidant activity versus S/F value are presented. The same profile of extraction found by the propolis in this study was also identified by other natural samples using supercritical CO_2_ such as for obtaining tagitinine C from *Tithonia diversifolia* [77], peach (*Prunus persica*) [78] and for cocoa beans [79].

**Table 1:** Determination of the pilot kinetic of extraction for green propolis with accumulated extract mass yield (%), total phenolic content yield (%) and antioxidant activity (%) by each experiment obtained (mean ± standard deviation) (Conditions: 40°C, 100 bar, CO_2_ flux of 6 g/min and 7.5 g green propolis).

Number of experiment	Time (min)	Volume of CO_2_ (m^3^)	S/F	Mass of Extract (Accumulated yield %)	Total Phenolic (Accumulated yield %)	Antioxidant activity (Accumulated yield %)
**1**	8.68	0.030	7.28	0.8346±0.0650	11.56±0.43	16.39±0.81
**2**	18.39	0.030	14.56	1.2073±0.1329	19.97±0.72	26.01±0.91
**3**	29.43	0.030	21.84	1.4086±0.1404	23.97±0.60	31.55±0.83
**4**	45.68	0.045	32.76	1.6146±0.0678	29.11±0.41	37.99±0.60
**5**	58.68	0.045	43.68	1.7206±0.0329	32.27±2.05	41.43±0.49
**6**	78.50	0.060	58.25	1.8926±0.0782	37.35±2.19	47.51±0.47
**7**	100.50	0.060	72.81	2.0573±0.1989	43.47±0.77	53.89±0.42
**8**	121.50	0.060	87.37	2.1933±0.2564	49.52±1.31	59.46±0.76
**9**	150.50	0.090	109.22	2.3960±0.3661	56.71±2.02	67.53±1.30
**10**	187.50	0.090	131.06	2.5373±0.4091	61.27±2.35	73.09±1.71
**11**	224.50	0.090	152.91	2.6846±0.4440	66.76±2.57	79.04±1.94
**12**	252.50	0.090	174.75	2.8286±0.5383	73.49±3.18	85.68±2.56
**13**	288.50	0.090	196.60	2.9720±0.5977	79.67±3.65	92.80±3.01

After analysing the behaviour of extraction using supercritical CO_2_ under the conditions determined for the propolis and obtaining the global curves of extraction, the value of 110 was identified as the preferred S/F (Fig 3). At this point, a yield of 0.129±0.01% was determined for the quantity of extract obtained. A low yield was already expected in the process of propolis extraction by SFE, as it has been reported by other studies [24,38,80–81]. Furthermore, as described by Catchpole et al. [24], propolis is not very soluble in supercritical CO_2_, but it is much more soluble in a mixture of CO_2_ + ethanol. For that reason, the lack of a co-solvent with polar characteristics can implicate in a low yield in this phase of the process.

Different authors reported as the best extraction S/F the one corresponding to the global curve phase (of yield in extract mass) between the end of the increasing rate of extraction and the start of the decreasing rate [48,82–83]. The extraction curve of a natural matrix using supercritical fluids is not a linear function of time [82,84]. The curve presented in Fig 4A represents a pattern to extract natural products, also identified for the sample of black pepper (*Piper nigrum*, L.) by Ferreira and Meireles [85], for the sample of propolis by Biscaia and Ferreira [25] and for the sample of annatto (*Bixa orellana* L.) by Albuquerque and Meireles [48]. An initial linear part was identified (constant extraction rate = CER), followed by a decreasing rate and ending at a near zero extraction rate. The period determined as CER is that in which exists the presence of an easily accessible solute on the surface of the matrix particles. The resistance to the mass transference is primarily on the external region of the particle [25,82,85–87]. In this situation, the process of mass transference is controlled by convection (solvent flow). In standard models of an extraction global curve for supercritical fluids there are three typical phases: (i) constant extraction rate (CER); (ii) falling extraction rate (FER); and (iii) diffusional region (DF). In general, 50 to 90% of the total extractable material is obtained in the constant region of extraction rate, and the optimization process should be focused on this region [82]. Albuquerque and Meireles [48] also performed preliminary kinetic assays to select the parameters of the process which are more adequate to determine the GYIs, when they analysed annatto (*Bixa orellana* L.) and identified 35 as the best value of S/F for the studied sample.

**Fig 5:**
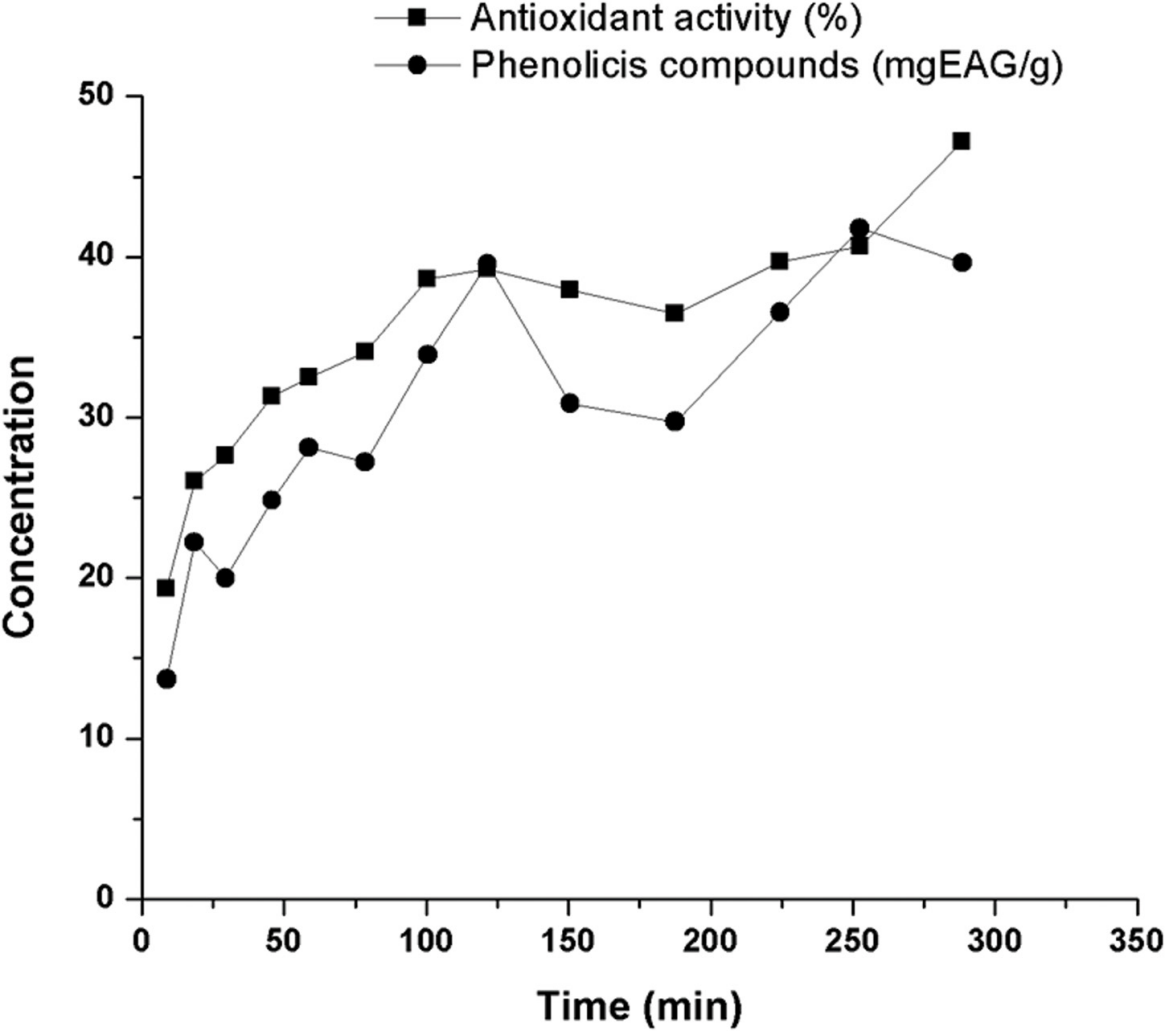
Profile of extraction of green propolis in relation to the content of phenolic compounds and antioxidant activity obtained at different times of extraction to determine the pilot kinetic curve (Conditions: 40°C, 100 bar, CO_2_ flux of 6.0 g/min and 7.5 g green propolis).

Since one of the objectives of this study was to define the conditions of the process for a better and greater extraction of the propolis bioactive compounds such as the antioxidant compounds (polyphenols); the global extraction curves for yield in total phenols and antioxidant activity (Fig 4B) were determined. This was aimed at verifying the content of the compounds obtained according to the S/F employed, also considering the time of extraction. It is possible to identify on the Fig 4B that, where the S/F value is 110, there is approximately 60% of the extractable phenolic compounds (in the conditions used). It was observed that by increasing the S/F to 150, the yield of these compounds increases 8%, what may not justify the time and solvent (CO_2_) expenditure and other costs in the process (if chosen the S/F higher than 110). In addition, the S/F of 110 was determined in the CER, which as reported in different studies, it is represented by a constant region extraction, and further where the and further where the solute is easily accessible on the surface of the matrix particles, facilitating the extraction process [82,84,87]. On Fig 5, the profile of extraction in relation to the content of total phenolic compounds and antioxidant activity is presented (not accumulated values).

### Determination of co-solvent concentration

After the kinetic assays were performed, the experiments were developed to determine the percentage of co-solvent (m/m) to compound the extraction system and afterwards, it selects the process best parameters to determine the GYIs. The polyphenols are the main components of interest in propolis, which has several hydroxyl groups. They are hydrophilic molecules, due to this characteristic, methanol, ethanol and water have been used as solvents in its extraction. Solvents such as ethyl acetate, have also been used, and also aqueous mixtures of methanol, ethanol or acetone are often the choice of solvents for the recuperation of a wide range of phenols from several types of samples [88]. The ethanol was selected as a co-solvent due to the different studies showing that the presence of this component intensifies the extraction of the compounds of a less lipophilic nature in propolis, increasing the yield of the extractive process with CO_2_ and it obtains relevant compounds such as Artepillin C (HPPC) and p-coumaric acid [2,24,36,38,49]. Table 2 shows the results for the content of phenolic compounds, flavonoids and IC_50_ (DPPH) using 1 and 2% ethanol (co-solvent) in relation to the CO_2_ mass (m/m), and without it. Also, it shows the concentration of Artepillin C and p-coumaric acid in the presence of a co-solvent.

**Table 2:** Determination of the content of phenolic compounds, flavonoids, IC_50_ (DPPH), 3,5-diprenyl-4-hydroxicinnamic acid (Artepillin C) and acid 4-hydroxycinnamic (p-coumaric acid) of the green propolis extracts obtained by supercritical extraction with CO_2_ and co-solvent in different concentrations (50°C, 250 bar and CO_2_ flux of 6 g/min).

Parameters co-solvent (m/m)	Analyses				
	Total Phenolic (mgEAG/g)	IC _50_ (μg/ml)	Flavonoids (mg EQ/g)	HPPC (μg/mL)	P-coumaric acid (μg/mL)
**0% ethanol**	62.21±1.12^a^	201.98±2.23^a^	20.27±0.71^a^	—	—
**1% ethanol**	80.3±1.68^b^	145.25±3.62^b^	26.53±0.67^b^	546.89±20.09^a^	34.82±2.01^a^
**2% ethanol**	66.72±1.49^c^	193.75±4.27^c^	23.02±0.47^c^	308.47±13.24^b^	14.32±1.24^b^

Values representing the same letter, on the same column, do not show significant differences (p>0.05) by the Tukey Test at 95% confidence.

The addition of a co-solvent in the supercritical carbon dioxide can significantly improve the extraction [89]. From the results shown on Table 2, it can be demonstrated that the presence of the co-solvent on the SFE process increases the extraction capacity of the phenolic compounds, flavonoids and antioxidant activity (IC_50_) of the green propolis under the conditions used. The results for these parameters are significantly better on extracts using 1% ethanol, when compared to the process using 2%, or to its total absence. Furthermore, the concentration of Artepilllin C and p-coumaric acid was significantly superior with the presence of 1% ethanol in the system (m/m) (Table 2). Lee et al. [2] investigated the use of different concentrations of ethyl acetate (2 to 6% m/m) as a co-solvent to increase the efficiency of the extractive process of the Artepillin C in propolis samples. The best results were identified with the addition of 6% of co-solvent. The difference between the literature and the present work is, probably, due to differences in the extraction method and the characteristics of the raw material. Lin et al. [89] identified that the quantity of extractable oil from *Schisandra chinensis* seeds are significantly increased with the presence of ethanol as co-solvent. The assumption is that the role of the co-solvent in the extraction is, mainly, to decrease the thickness of the limit layer for mass transfer, considering that the increase in the quantity of ethanol in the system does not significantly increase the process yield.

A possible explanation for the improved extraction capacity with the presence of a lower quantity of ethanol (1% m/m) is that this substance has, in its molecules, the group OH, which make them capable of forming hydrogen bonds among themselves. On the other hand, the molecules of the polar components present in propolis also form hydrogen chains among themselves. Therefore, in order to the solute to be solvated by the solvent, the formation of new hydrogen bond is necessary, this time between the ethanol molecules and the molecules of the polar compounds of the propolis. The energy required to form these new connections is from the rupture between the hydrogen bonds and the solute molecules. However, when the quantity of co-solvent (% ethanol) is too high, there will not be enough energy to break the bonds between the ethanol molecules, and consequently less polar compounds will be solubilized by the solvent, causing a decrease in the extraction yield of these compounds and of the antioxidant activity [90].

Other studies evaluated the yield of the extraction process, depending on the percentage of co-solvent used in the system, and identified that, depending on the increase on the mass percentage of the co-solvent, there is no significant increase–and there may even be a decrease–on the quantity of the extract obtained. This, consequently, reduces the extraction of the relevant compounds in the matrix. Biscaia and Ferreira [25] compared the extraction yields of the propolis obtained through different procedures, including the extraction with supercritical CO_2_ with (2.5 and 7%) and without the presence of ethanol (co-solvent). The maximum yield for the supercritical extraction process was obtained using intermediate conditions of ethanol (5% m/m). Marqués et al. [91] determined the best results for the extraction of antioxidant compounds of grape seeds by SFE (CO_2_ supercritical), when used an approximate concentration of 3% (m/m) ethanol. Samples of Brazilian propolis and propolis from Taiwan were studied, using supercritical CO_2_ and CO_2_ with the addition of co-solvent (ethanol and water, 1:2 and 1:5 (m/v)). This was aimed at increasing the solubility in water and the anti-cancerous capacity of the extracts [49]. The addition of ethanol (1:5 m/v) in the process increased the extraction capacity of the nine phenolic compounds identified and quantified in the samples, whereas the addition of water did not show a positive influence in the process, when compared to extraction with only CO_2_. Some patents also revealed that the propolis extracts obtained by SFE with CO_2_ and ethanol improved the extraction efficiency of active compounds from this matrix, including, for example, the Artepillin C and p-coumaric acid [92–93]. Similar results to this study were identified by Garmus et al. [94], Porto et al. [95], Bitencourt et al. [96] and Campos et al. [97], when evaluated different concentrations of the co-solvent in the extraction process.

### Determination of global yield isotherms (GYIs)

**Fig 6:**
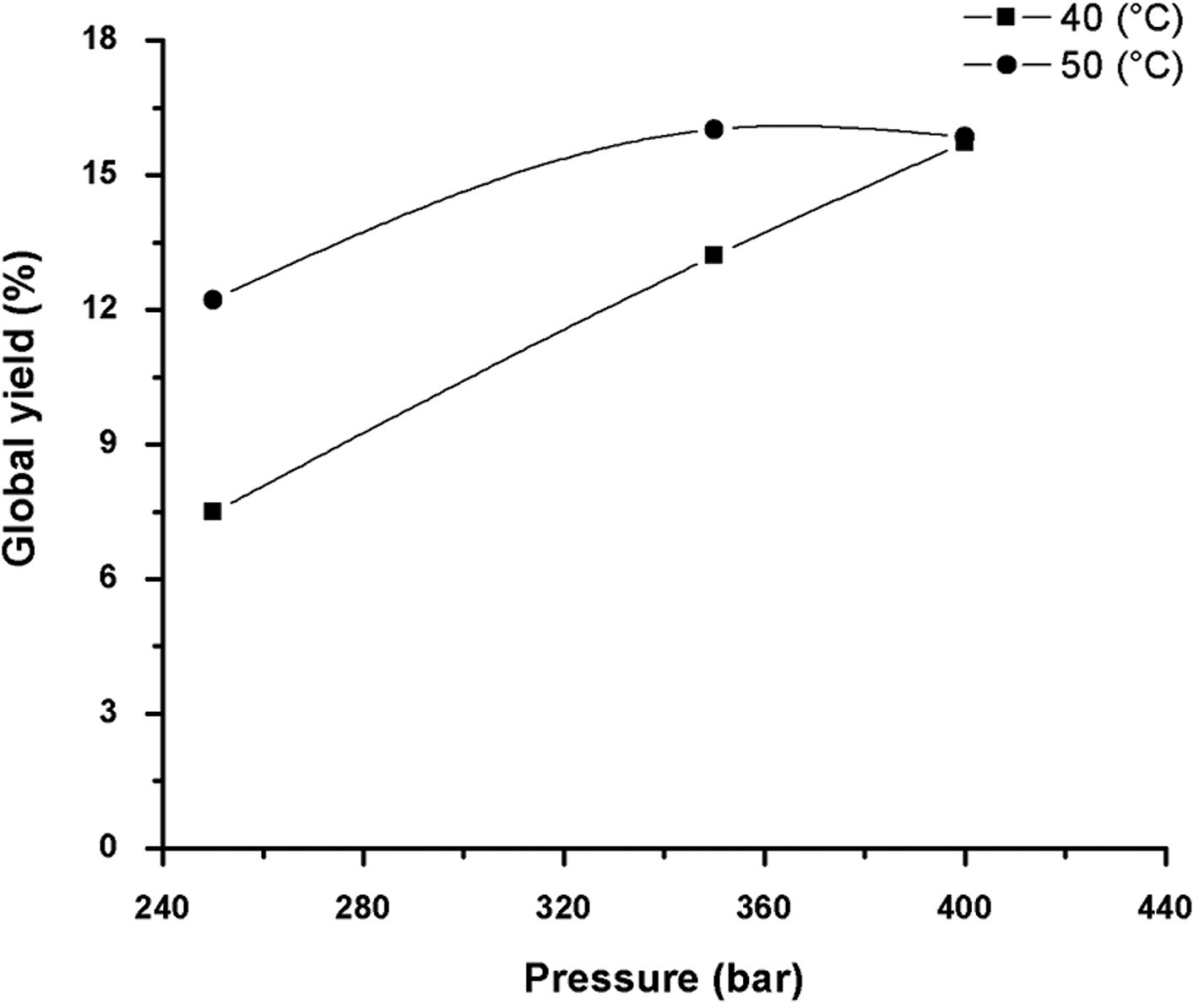
Global Yield Isotherm (%) for green propolis using CO_2_ as supercritical fluid, ethanol as co-solvent (1% m/m) at temperature of 40 and 50°C and pressures of 250, 350 and 400 bar (CO_2_ flux of 6.0 g/min; extraction time 2 h 30 min).

In order to elaborate the GYIs, the best conditions of previous assays were used. That is, S/F value of 110, 1% co-solvent (ethanol m/m), CO_2_ flow of 6.0g /min with pressure variations (250, 350 and 400 bar) and temperature (40 and 50°C). On Fig 6, the GYI for the extraction process of the green propolis are shown using the different conditions.

Under the pressures of 250 and 350 bar, the yield of the extraction process is significantly higher at temperature of 50°C when compared to 40°C. In these conditions the density of the solvent decreases considerably with the temperature, conducting to a lower solvation and also to a lower global yield. At 400 bar, the two isotherm curves crossover. At this point, there is no influence of the temperature on the global yield. This behaviour is, probably, an indication of the vicinity of the isotherm crossover, and was also identified by different studies reporting the competition between the two effects caused by the increase in temperature: (1) the reduction in the solvent power, with a decrease in yield, and (2) an increase in the pressure of the solute vapour, with an increase in yield [25,98–100]. In the study peformed with the Brazilian propolis at 200 bar, the yield was constant for all tested temperatures (30; 40; and 50°C) [25].

For the isotherm obtained at 40°C, it was observed that the global yield increases with the increase in pressure, whereas for the isotherm at 50°C, the global yield decreases with pressures above 350 bar. Therefore, there is no increase in power of extraction with the increase in vapour pressure at this point. On the isotherm at 40°C, the increase in pressure accelerates the mass transfer in the supercritical extractor, and increases the yield of extraction for the propolis components [36].

Therefore, the increase in density (which increases with pressure) increases the solvation power of the supercritical fluid, as well as the increase in temperature improves its diffusivity, the transfer of mass and consequently the extraction capacity [28]. However, high temperatures are not always advantageous, since they result in the reduction of the fluid’s density, decreasing its solvent power [101]. For this reason, the definition of the adequate binomial pressure/temperature determines the success or otherwise of the extractive process. According to Meireles [102], the importance of measuring the global yield is due to the fact that this is an efficient methodology to select the best pressure and temperature conditions, according to the yield obtained in an initial phase of development in the supercritical extraction process. Therefore, for the raw material studied, the best global yield was obtained at pressure of 350 bar and temperature at 50°C (Fig 6).

Piantino et al. [103] identified the best global yield for leaves of *Baccharis dracunculifolia* at the higher temperature and pressure used (60°C and 400 bar), whereas the lowest global yield was obtained at lower conditions (40°C and 200 bar). Lin et al. [89] determined the pressure of 350 bar and temperature of 50°C as the best conditions applied for the extraction of *Schisandra chinensis* seeds oil. Besides, they observed the same behaviour identified for the green propolis, that is, when the temperature increases from 40 to 50°C, with constant pressure of 350 bar, there is a significant increase in the extraction yield.

**Fig 7:**
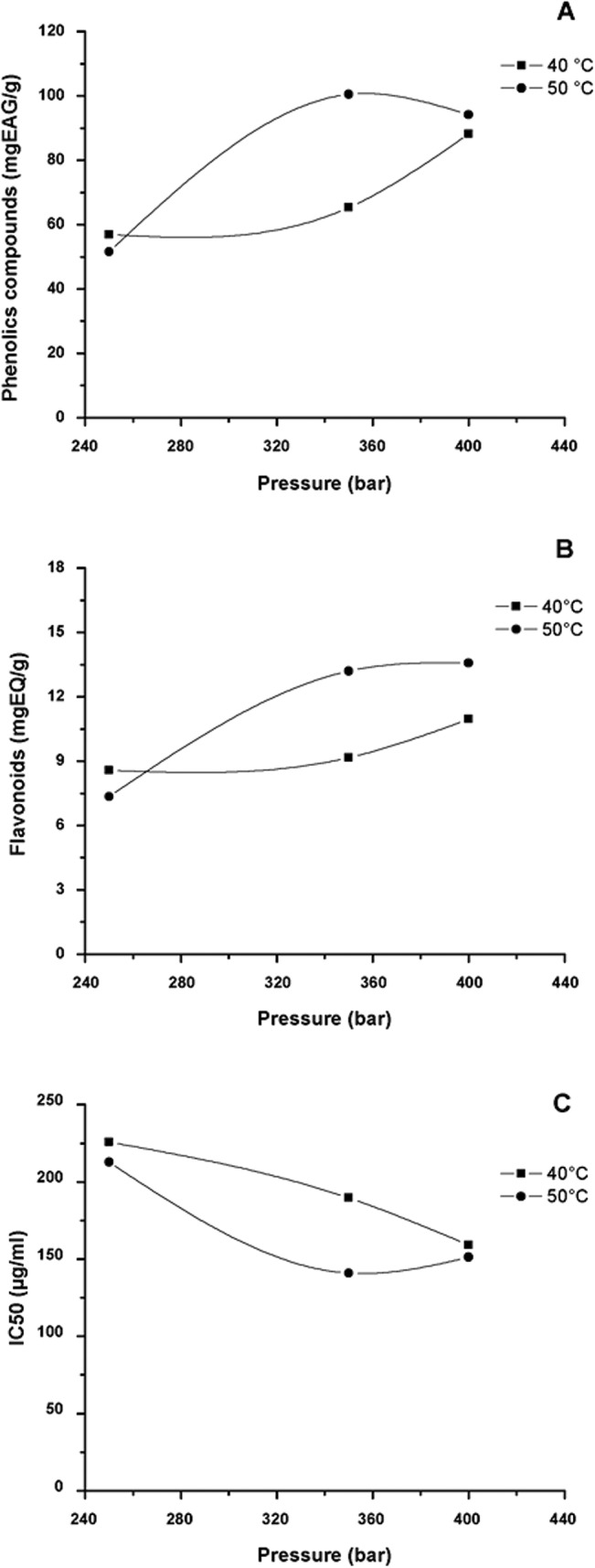
Isotherms for (A) total phenolic compounds; (B) flavonoids; and (C) antioxidant activity (IC_50_) for extraction of green propolis using CO_2_ as supercritical fluid, ethanol as co-solvent (1% m/m) at temperatures of 40 and 50°C and pressures of 250, 350 and 400 bar (CO_2_ flux of 6.0 g/min; extraction time 2 h 30min).

On Fig 7, the isotherms for the yield of total phenolic compounds, flavonoids and antioxidant activity (IC_50_) obtained under the different conditions analysed are showed. They present a similar profile to the global yield isotherm (Fig 6). The best results for the studied parameters were presented on the isotherm at 50°C, with a significant increase in the extraction of phenolic compounds, flavonoids and antioxidant activity (IC_50_), when the pressure is increased from 250 to 350 bar. The increase in temperature at a fixed pressure reduces the density of the CO_2_ in the supercritical state, reducing solubility. However, at the same time, it increases the vapour pressure of the compounds to be extracted. In this way, there is an increase in the tendency to turn these compounds into a fluid phase.

According to what was shown by the global yield isotherm at 50°C, the increase in pressure to 400 bar also represents a significant reduction in the quantification of the phenolic compounds and a reduction in the antioxidant activity. For the extraction of flavonoids, keeping the temperature constant at 50°C, the increase in pressure from 350 to 400 bar does not significantly influence the extraction yield of these compounds. This is probably related to the increase in solubility of the flavonoids in supercritical CO_2_ at a higher pressure [104–106]. For the isotherm at 40°C, the increase in pressure increases the concentration of the compounds analysed and the antioxidant activity. This could be justified according to the fact that the solubility of the solute increases with the operation pressure at a constant temperature, due to the increase in the solvent density [37]. These results point to a temperature of 50°C and pressure of 350 bar as ideal conditions for the extraction of the propolis bioactive compounds.

It should be highlighted that the flavonoids are considered the most important compounds of propolis for determining the quality of this material. Zordi et al., [36], analysed multiple regression for obtainment of polyphenols in propolis extracts using supercritical CO_2._ Considering the content of total flavonoids, they identified that the parameters of pressure and temperature had significant effects, where the higher yields for these compounds (>10%) were observed at 270 and 200 bar at 40°C. You et al. [49] evaluated the extraction of flavonoids in propolis samples and identified that the extraction yield increased with pressure (276–345 bar), with the best yield point (10,5% w/w) being obtained under the conditions 345 bar and 45°C, very close to those conditions used in this study.

**Fig 8:**
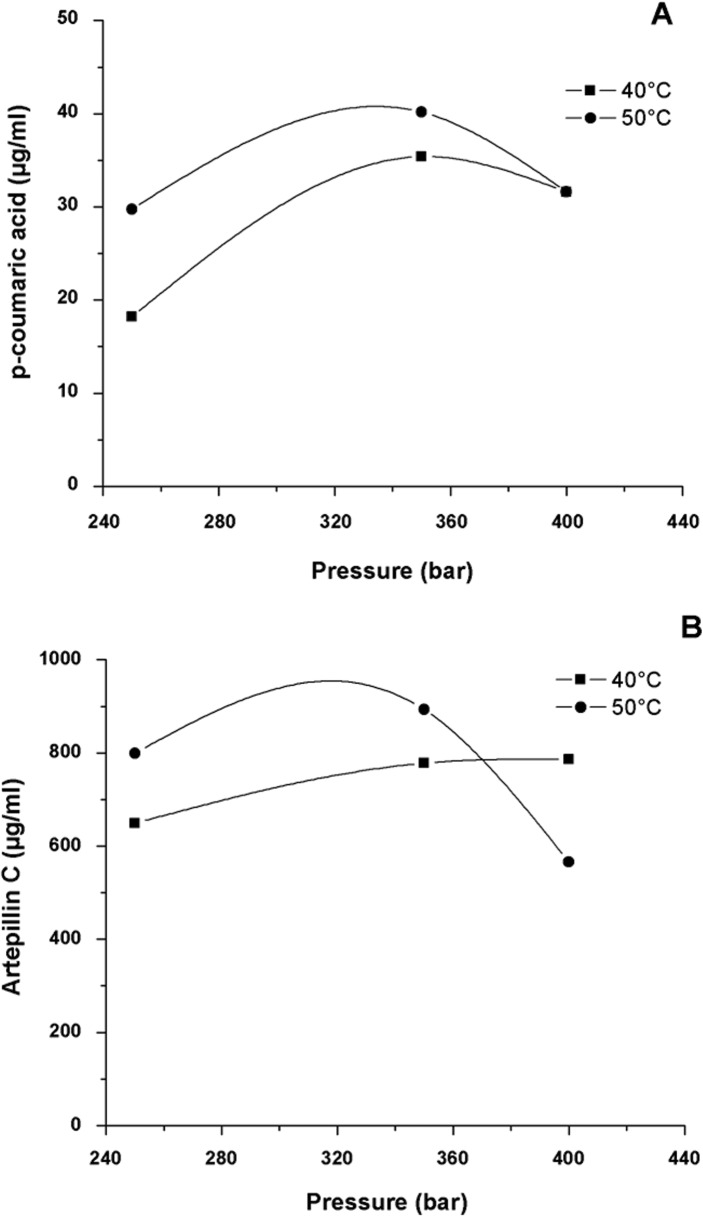
Isotherms for (A) concentration of p-coumaric acid; and (B) Artepillin C; on green propolis extracts obtained using CO_2_ as supercritical fluid, ethanol as co-solvent (1% m/m) at temperatures of 40 and 50°C and pressures of 250, 350 and 400 bar (CO_2_ flux of 6.0 g/min; time of extraction 2 h 30 min).

The influence of pressure and temperature for the extraction of p-coumaric acid and Artepilin C (μg/ml) are presented in Fig 8. On Table 3 are expressed the results obtained for the concentration of these two markers in μg/ml and g/100g for each extract, and also the results for the global yield (S6 Fig). These results identified these two markers of green propolis according to the isotherms presented on Figs 6 and 7.

**Table 3:** Determination of the acid 4-hydroxycinnamic (p-coumaric acid) content and 3,5-diprenyl-4-hydroxicinnamic acid (Artepillin C) and global yield values of the green propolis extracts obtained by supercritical extraction at different temperatures and pressures (mean ± standard deviation).

Conditions of Temperature (°C) and Pressure (bar)	P-cumaric Acid (μg/ml)	P-coumaric Acid (g/100g)	Artepillin C (μg/ml)	Artepillin C (g/100g)	Global yield (%)
T = 40 P = 250	18.20±0.63^a^	0.18±0.01^a^	649.10±6.69^a^	6.49±0.06^a^	7.48±0.12^a^
T = 50 P = 250	29.76±3.59^b^	0.29±0.03^b^	799.32±3.46^b^	7.99±0.03^b^	12.22±0.13^b^
T = 40 P = 350	35.39±0.55^c^	0.35±0.01^c^	778.04±1.32^c^	7.78±0.13^c^	13.02±0.07^c^
T = 50 P = 350	40.18±5.26^d^	0.40±0.05^d^	893.08±1.62^d^	8.93±0.01^d^	16.02±0.53^d^
T = 40 P = 400	31.60±2.46^b^	0.31±0.02^b^	786.23±8.16^b^	7.86±0.08^b^	15.73±0.14^e^
T = 50 P = 400	31.62±3.84^b^	0.31±0.03^b^	565.92±7.09^e^	5.66±0.07^e^	15.85±0.21^e^

Values showing the same letter, in a same column, do not present significant differences (p>0.05) according to Tukey Test at 95% confidence.

For the p-coumaric acid (Fig 8A), the behaviour is the same presented by the global yield isotherm (Fig 6) at pressure 400 bar, where both isotherm curves crossover. There is, however, no influence of temperature on the extraction yield of this compound (to 400 bar pressure). In the two isotherms, an increase in concentration of the p-coumaric acid is observed when the pressure is increased from 250 to 350 bar. Besides that, the increase in temperature represents an increase in the yield of the compound at the constant pressures of 250 and 350 bar, justified through the increase in the vapour pressure, considering the simple effect. For Artepillin C, a similar behaviour to that of the p-coumaric acid is observed with the increase in temperature and pressure up to 350 bar. That means there is an increase in concentration of the compound. In higher pressures (400 bar), the concentration of the Artepillin C decreased with the increase temperature. In the other hand, in lower pressures (250 and 350 bar), the opposite behaviour was observed, it means that the concentration increases with the increase temperature and constant pressure. This type of behaviour can be explained based on the effect of temperature and pressure on the variation in density of the solvent, and due to the effect of temperature on the vapour pressure of the solute [81].

The isotherms crossover at 370 to 375 bar (Fig 8). In this region, the effect of temperature on the increase of vapour pressure compensated for the effect of temperature on the decrease in density of the solvent. Due to the existence of this point, the lower and higher concentrations occurred in the same isotherm of 50°C. For the bixin samples, Silva et al. [107] determined the crossover pressure at 280 bar, whereas Albuquerque and Meireles [48] identified it at 200 bar. A similar behaviour to that of this study for the extraction of Artepillin C and p-coumaric acid was identified by Piantino et al. [103], using the conditions of 300 bar and 40 and 50°C, and by Paviani et al. [81] using the conditions of 240 bar and 20, 35 and 50°C. That shows that the increase temperature promotes a higher yield of these compounds at a determined constant pressure.

The phenolic compound Artepillin C (HPPC), with confirmed important biological activities, was present in higher concentration in the extracts obtained at 50°C and 350 bar, showing 8.93±0.01 g of the compound in 100 g of propolis extract. Values inferior to those used in this study for Artepillin C content were found by Chen et al. [38] (1.27 g/100g) in samples of Brazilian propolis by SFE, under the conditions of 207 bar, 60°C and 6% ethyl acetate co-solvent; by Biscaia and Ferreira [25] (0.457 g/100g) under the conditions 150 bar, 40°C and 5% ethanol as co-solvent; and by Paviani et al. [81] (0.524 g/100g) under the conditions of 250 bar, 50°C and 5% of ethanol as co-solvent. The variations found are related to the origin of the matrix [108], as well as the conditions of process used.

Finally, it is demonstrated that the effect of pressure on the kinetics of extraction using supercritical CO_2_ is already well established in the literature. There is no consensus within the scientific community to support that an increase in the operating pressure would have a positive effect in the extraction rate of biologically active compounds, considering the behaviour of each sample [109–110]. However, if the increase in pressure acts positively, increasing the extraction capacity to a constant temperature, the financial viability to work at a high pressure has to be analysed case by case, as any increase of pressure is directly related to an increase of energy consumption.

In the case of obtaining biologically active compounds from green propolis, working with high pressures does not seem economically convenient, considering that the extraction capacity of the relevant compounds is decreased. This is especially true for Artepillin C, the main marker for green propolis [38,103], and presently considered as a compound of great interest to the pharmaceutic industry.

**Fig 9:**
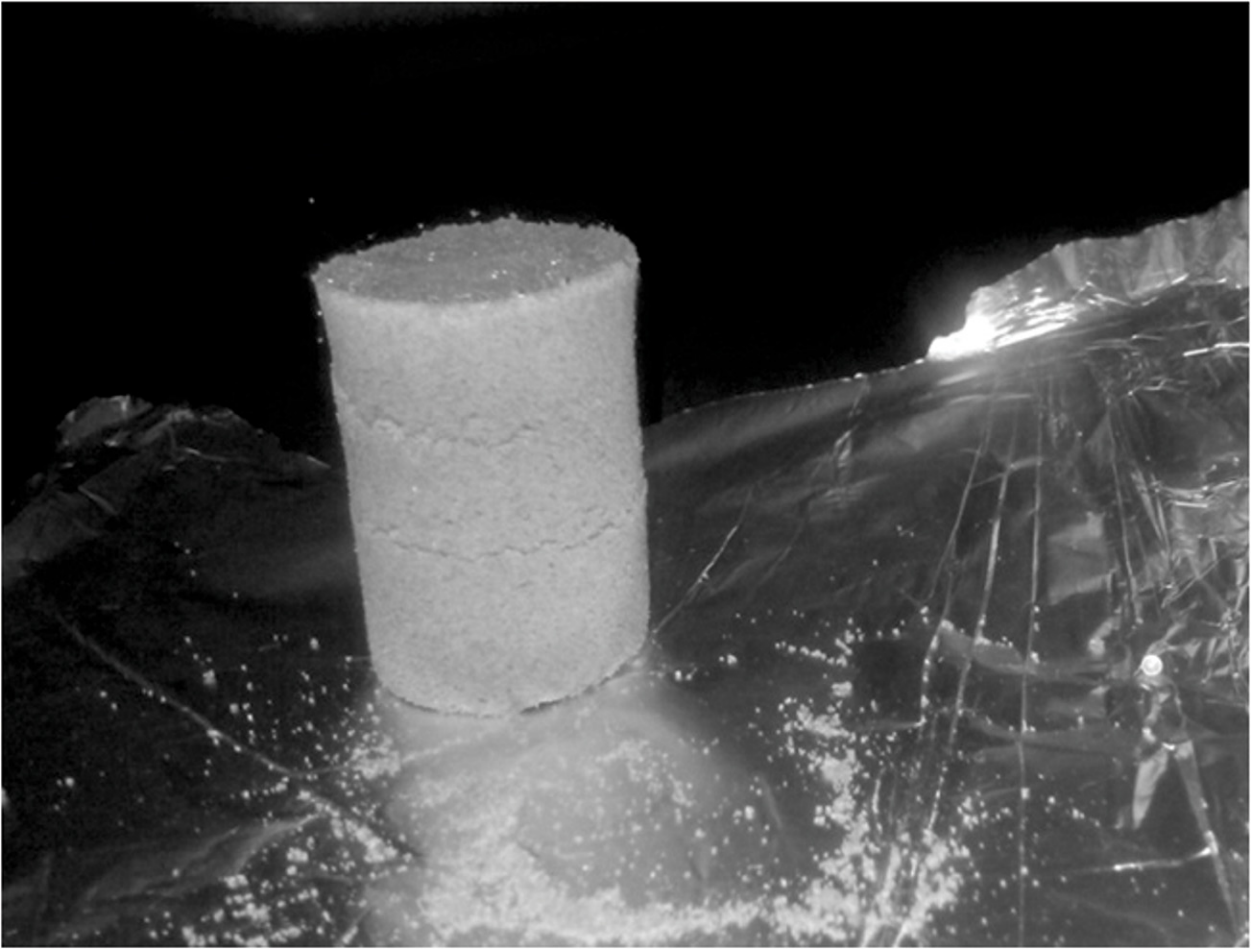
Raw propolis sample obtained after supercritical extraction using CO_2_ and ethanol as co-solvent.

On Fig 9, an image of propolis sample after SFE with CO_2_ and ethanol as co-solvent is shown. It is consensus in the literature that extracts obtained by SFE present a different profile when compared to that obtained by conventional extractions, such as the alcoholic extraction or that by Soxhlet [2,10,38,111]. For propolis, despite a lower yield when compared to alcoholic extraction, the extracts obtained by SFE are considered as highly valuable products, considering the profile of compounds shown. According to Biscaia and Ferreira [25], the viability of the process is directly related to the quality of the final product, in order to improve the biological potential of the raw material. Lee et al. [2] extracted Artepillin C from the Brazilian green propolis samples using supercritical CO_2_ modified with a co-solvent, followed by chromatography in column, in order to obtain very pure Artepillin C. Furthermore, the propolis residue, after extraction by SFE, can be used for the traditional extraction, producing a good quantity of flavones and phenols [36].

## Conclusion

This study presented important aspects regarding the process parameters for obtaining green propolis extracts using supercritical CO_2_. Considering that propolis is a complex material, the use of extraction with supercritical fluids, especially CO_2_, is extremely attractive to obtain extracts with high added value, despite the low yield of the process. In this study, it was identified that the addition of co-solvent in the system is an advantage to improve the extraction process of the biologically active compounds present in the green propolis, due to the hydrophilic characteristic of the active compounds. The best extracts were obtained in the conditions of 50°C, 350 bar, SF 110 and the presence of 1% ethanol as co-solvent. In these conditions, the extracts have a high content of phenolic compounds, high antioxidant activity (represented by the low IC_50_ obtained), as well as a high flavonoid content. The concentration of Artepillin C obtained in these conditions is very important, which is demonstrated by the obtainment of extracts with important biological applications, already evidenced in other studies, as well being as a product of interest for food, pharmaceutic and cosmetic industries.

## Supporting Information

S1 Fig The equipment used to obtain the extracts of green propolis was a pilot unity called SFT-110 Supercritical Fluid Extractor (Supercritical Fluid Technologies, Inc.). (DOCX)

S2 Fig Mounting the extraction cell (100 ml capacity): The ethanol was homogenised with the sample and placed in the extraction cell, together with wool and glass pearls to fill the cell. (DOCX)

S3 Fig Green propolis extracts obtained by supercritical extraction. (DOCX)

S4 Fig Ultraviolet spectrum to the p-coumaric acid standard at 290 nm by HPLC. (DOCX)

S5 Fig Ultraviolet spectrum to the Artepillin C standard at 290 nm by HPLC. (DOCX)

S6 Fig Chromatogram Green propolis extract obtained by supercritical extraction in the conditions of 50°C, 350 bar. (DOCX)

S1 Table Extraction for green propolis in relation to the Mean DPPH (%) and Mean Total Phenolic (%) obtained at different times for obtaining the pilot kinetic (Conditions: 40°C, 100 bar, CO_2_ flux of 6 g/min and 7.5 g of green propolis). (Table support of Fig 3).
(DOCX)
