# Supplementary material for: Determination of Parameters for the Supercritical Extraction of Antioxidant Compounds from Green Propolis Using Carbon Dioxide and Ethanol as Co-Solvent
Source: PLoS One. 2015 Aug 7;10(8):e0134489. doi: 10.1371/journal.pone.0134489 (PMC4529176; doi:10.1371/journal.pone.0134489)
Supplement: S1 Fig — (DOCX) [file pone.0134489.s001.docx]

**
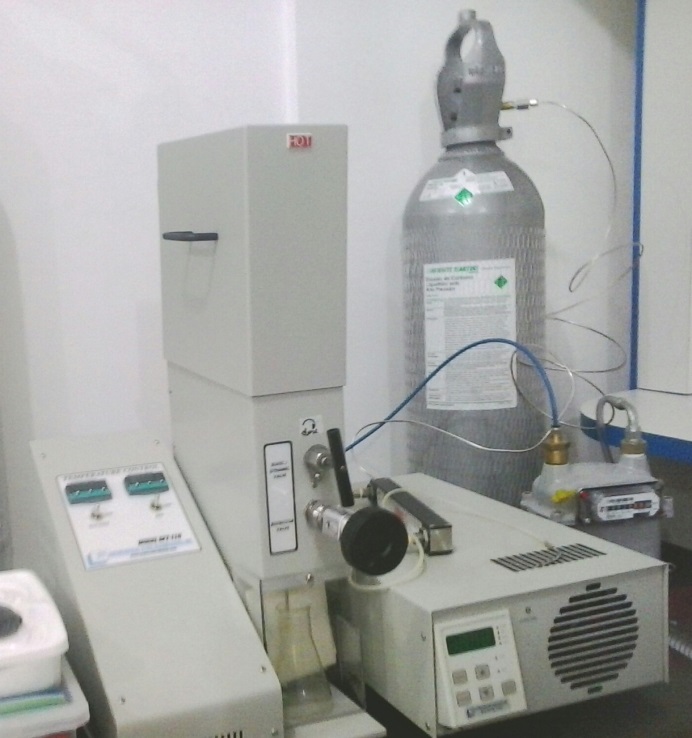
**

S1 Fig. The equipment used to obtain the extracts of green propolis was a pilot unity called SFT-110 Supercritical Fluid Extractor (Supercritical Fluid Technologies, Inc.).
