# Supplementary material for: Determination of Parameters for the Supercritical Extraction of Antioxidant Compounds from Green Propolis Using Carbon Dioxide and Ethanol as Co-Solvent
Source: PLoS One. 2015 Aug 7;10(8):e0134489. doi: 10.1371/journal.pone.0134489 (PMC4529176; doi:10.1371/journal.pone.0134489)
Supplement: S2 Fig — (DOCX) [file pone.0134489.s002.docx]

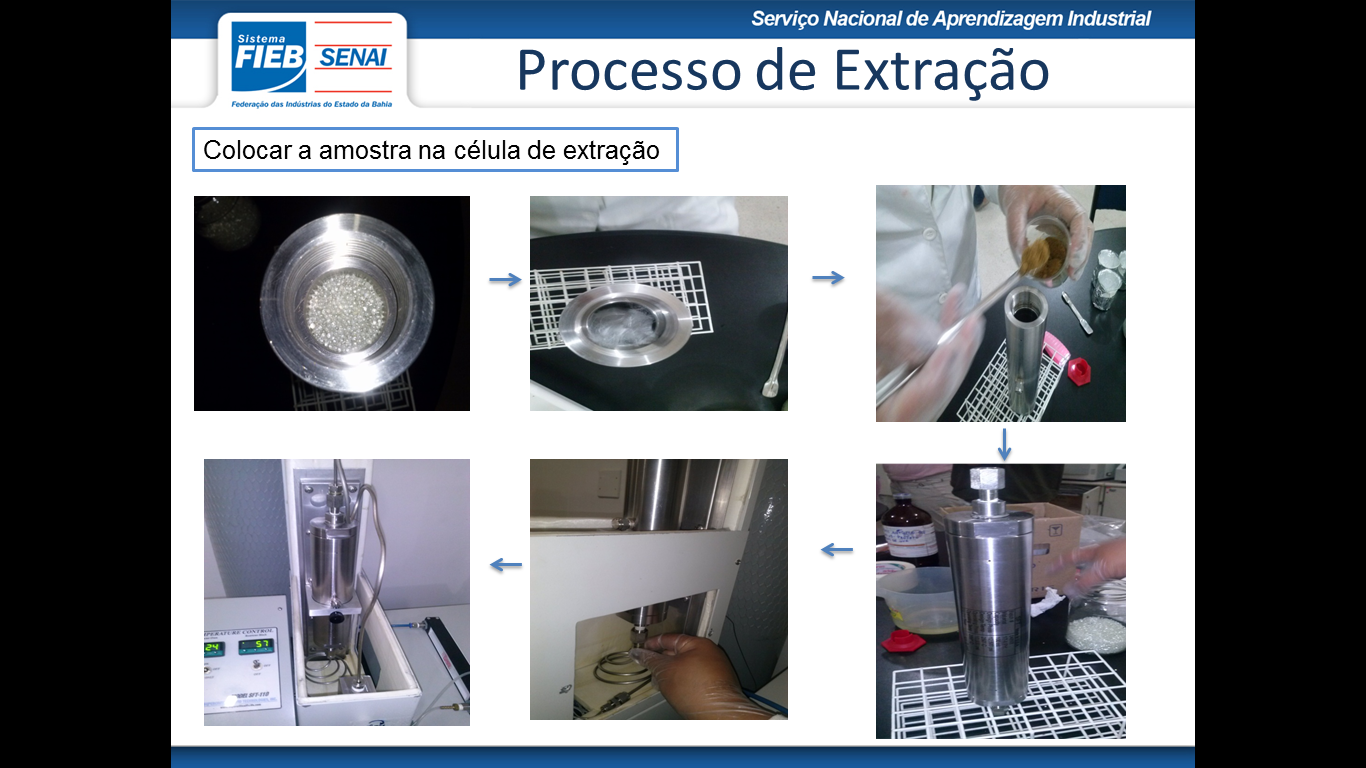


S2 Fig. Mounting the extraction cell (100 ml capacity): The ethanol was homogenised with the sample and placed in the extraction cell, together with wool and glass pearls to fill the cell.
