# Supplementary figures and images for: Determination of Parameters for the Supercritical Extraction of Antioxidant Compounds from Green Propolis Using Carbon Dioxide and Ethanol as Co-Solvent
Source: PLoS One. 2015 Aug 7;10(8):e0134489. doi: 10.1371/journal.pone.0134489 (PMC4529176; doi:10.1371/journal.pone.0134489)

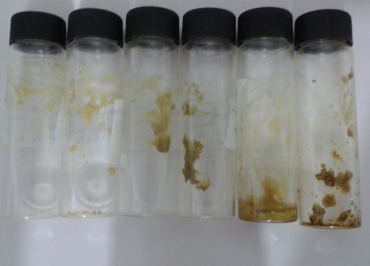


S3 Fig. Green propolis extracts obtained by supercritical extraction.

Supplement: S3 Fig — (DOCX) [file pone.0134489.s003.docx]
