# Supplementary material for: Determination of Parameters for the Supercritical Extraction of Antioxidant Compounds from Green Propolis Using Carbon Dioxide and Ethanol as Co-Solvent
Source: PLoS One. 2015 Aug 7;10(8):e0134489. doi: 10.1371/journal.pone.0134489 (PMC4529176; doi:10.1371/journal.pone.0134489)
Supplement: S4 Fig — (DOCX) [file pone.0134489.s004.docx]

S4 Fig. Ultraviolet spectrum to the p-coumaric acid standard at 290 nm by HPLC.
