# Supplementary material for: Determination of Parameters for the Supercritical Extraction of Antioxidant Compounds from Green Propolis Using Carbon Dioxide and Ethanol as Co-Solvent
Source: PLoS One. 2015 Aug 7;10(8):e0134489. doi: 10.1371/journal.pone.0134489 (PMC4529176; doi:10.1371/journal.pone.0134489)
Supplement: S6 Fig — (DOCX) [file pone.0134489.s006.docx]

S6 Fig. Chromatogram Green propolis extract obtained by supercritical extraction in the conditions of 50°C, 350 bar.
