# Supplementary material for: Determination of Parameters for the Supercritical Extraction of Antioxidant Compounds from Green Propolis Using Carbon Dioxide and Ethanol as Co-Solvent
Source: PLoS One. 2015 Aug 7;10(8):e0134489. doi: 10.1371/journal.pone.0134489 (PMC4529176; doi:10.1371/journal.pone.0134489)
Supplement: S1 Table — (Table support of Fig 3). (DOCX) [file pone.0134489.s007.docx]

S1 Table. Extraction for green propolis in relation to the Mean DPPH (%) and Mean Total Phenolic (%) obtained at different times for obtaining the pilot kinetic (Conditions: 40°C, 100 bar, CO2 flux of 6 g/min and 7.5 g of green propolis). (Table support of Figure 3).

| **Time** | **Mean DPPH (%)** | **Mean Total Phenolic (%)** |
| --- | --- | --- |
| 8,68 | 19,31086958 | 13,69099195 |
| 18,395 | 26,0097048 | 22,23532621 |
| 29,43 | 27,60353439 | 19,98892847 |
| 45,685 | 31,29300396 | 24,84275216 |
| 58,68 | 32,46429991 | 28,13212028 |
| 78,5 | 34,11263008 | 27,20949264 |
| 100,5 | 38,61335717 | 33,90857161 |
| 121,5 | 39,21922055 | 39,56468021 |
| 150,5 | 37,95633896 | 30,85988896 |
| 187,5 | 36,45508113 | 29,73669009 |
| 224,5 | 39,67360928 | 36,55611181 |
| 252,5 | 40,65314066 | 41,7709637 |
| 288,5 | 47,17560177 | 39,6449087 |
